# Commentary: Interpersonal Coordination in Soccer: Interpreting Literature to Enhance the Representativeness of Task Design, From Dyads to Teams

**DOI:** 10.3389/fpsyg.2019.01093

**Published:** 2019-05-14

**Authors:** Vincent Gesbert, Denis Hauw

**Affiliations:** Research Center in Psychology of Health, Aging and Sport, Faculty of Social and Political Sciences, University of Lausanne, Lausanne, Switzerland

**Keywords:** interpersonal coordination, task representativeness, Soccer, sense-making activities, enactive approach

This commentary discusses and extends the ideas in the perspective paper, “Interpersonal coordination in soccer: Interpreting literature to enhance the representativeness of task design, from dyads to teams” (Santos et al., 2018). Our goal is to mention missing parts such as the athlete's experience and how athletes use it to adjust their activity to the interpersonal coordination dynamics.

Although not explicitly mentioned, the authors' interpretation of the literature was based on the ecological dynamics framework (e.g., Silva et al., 2013). Within this framework, interpersonal coordination is assumed to result from self-organizing processes that encompass interacting parts like the players, ball, and environment in soccer. To understand how interpersonal coordination emerges, is sustained or disrupted, and changes during performance, researchers record players' behaviors during competition. From these positional data, they analyze the behavioral dynamics of interpersonal coordination through collective variables (e.g., relative phase). Variations in these measures describe the influence of informational constraints—like ball displacement dynamics—on the process of interpersonal coordination. As behavioral dynamics show statistical consistencies, they are interpreted as factors of this coordination. These informational constraints are also defined as possibilities for controlling goal-directed activity with others or affordances (Gibson, 1979). These affordances are subsets of the spatio-temporal structure of light converging at the eyes (Seifert et al., 2018) that each player's perceptual system may or may not detect. As a reminder, the pedagogical outcome in a realistic learning environment is the athletes' placement such that their perceptual systems become increasingly sensitive to the spatio-temporal information that specifies affordances (e.g., Chow et al., 2016).

With this brief summary, we acknowledge our surprise that the authors cited Bourbousson, Sève and McGarry (2010b) study, yet overlooked other studies from Bourbousson and colleagues within the enactive framework of their research program (e.g., Bourbousson et al., 2010a, 2012; Bourbousson and Fortes-Bourbousson, 2016). These works are nevertheless of great interest and relevance to the topic of their paper. In this commentary, we therefore (re)present the theoretical assumptions of the enactive framework and suggest how data collected within this design might contribute to improving task design representativeness.

The enactive framework assumes that agents actively build meanings through their actions and interactions with the environment, enabling them to develop other understandings of an unfolding situation (e.g., Varela et al., 1991; Di Paolo et al., 2011). Meanings are thus not mere affordances in the environment that specify properties of the individual-environment relationship. Instead, they are outcomes of interactions between individuals acting and the dynamics of their environment. For instance, Bourbousson et al. (2012) characterized how five basketball players shared concerns in a real match situation. The concerns corresponded to what made sense simultaneously for each of the players in the situation, thereby delimiting their embedded activities (i.e., the environmental information they were sensitive to in order to act). This sensitivity to environmental information was linked to what was relevant to the players at a given instant in relation to their activity. In another example, a soccer player who wanted to make a quick counterattack on the opponent's goal was sensitive to the opponent's poor positioning and perceived an opportunity to forward pass to a teammate (Gesbert and Durny, 2017). Within this framework, the players were actively adjusting the conditions for their exchanges with the environment, highlighting the autonomy of their activity (Froese and Di Paolo, 2011). And as they adjusted their activity, this gave rise to an experience expressing that each was the author of his own actions (e.g., Buhrmann and Di Paolo, 2015). Such experience is not considered an epiphenomenon that reduces the player-environment interaction to sensorimotor adjustments in order to detect affordances. Instead, it describes “a coherent set of habitual embodied actions, feelings and sensations” (Hauw, 2018, p. 56) that accounts for the phenomenological fraction of the player-environment interaction (Thompson, 2007).

Special attention should therefore focus on how players experience their ongoing interactions and dynamically adjust their interpersonal coordination. Thus, recent studies have shown how soccer players use their experience to actively adjust activity to the collective behavior dynamics (Gesbert et al., 2017; Feigean et al., 2018) and how team members are sensitive to environmental information as they monitor the ongoing interpersonal coordination through the feeling of being together with others (Lund et al., 2012; Himberg et al., 2018). Notably, Lund et al. (2012) described how rowers are sensitive to the tension they feel in their movements as they mutually adjust their activity. The traditional methods are retrospective phenomenological interview techniques like the elicitation or self-confrontation techniques, which aim to access and describe agents' lived experience at the level of pre-reflective consciousness: What is he/she trying to do? What is drawing his/her attention? (Gesbert et al., 2017; Hauw, 2018; Rochat et al., 2018). To illustrate, Gesbert et al. (2017) performed an enactive phenomenological analysis to characterize the environmental information that soccer players were attuned to as they adapted their activity to contextual demands during competition.

To conclude, we suggest extending and enriching the behavioral perspective (how do athletes act in the world?) described by Santos et al. (2018) with a phenomenological perspective (how do athletes experience their engagement with the world?) in order to enhance task representativeness in soccer. As Seifert et al. (2016, p. 110) suggested: ”*the use of an ecological dynamics framework could be enriched by the analysis of performers' experience because it gives experiential meaning to the (…) behavioral patterns*.” By accessing the environmental information that players are sensitive to as they adjust their activity and by understanding how this information shapes players' sense-making processes (i.e., phenomenological data), coaches would be better equipped to manipulate task constraints and increase the degree to which athletes' behaviors during training tasks replicate those of competition. From this co-adaptive relationship between coaches' and players' experiences, new opportunities for acting and learning would emerge.

## Author Contributions

VG and DH co-wrote the manuscript.

### Conflict of Interest Statement

The authors declare that the research was conducted in the absence of any commercial or financial relationships that could be construed as a potential conflict of interest.
